# Association of Fecal Microbial Diversity and Taxonomy with Selected Enzymatic Functions

**DOI:** 10.1371/journal.pone.0039745

**Published:** 2012-06-28

**Authors:** Roberto Flores, Jianxin Shi, Mitchell H. Gail, Pawel Gajer, Jacques Ravel, James J. Goedert

**Affiliations:** 1 Infections and Immunoepidemiology Branch, Division of Cancer Epidemiology and Genetics, National Cancer Institute, Rockville, Maryland, United States of America; 2 Cancer Prevention Fellowship Program, National Cancer Institute, Rockville, Maryland, United States of America; 3 Biostatistics Branch, Division of Cancer Epidemiology and Genetics, National Cancer Institute, Rockville, Maryland, United States of America; 4 Institute of Genome Sciences, University of Maryland School of Medicine, Baltimore, Maryland, United States of America; Baylor College of Medicine, United States of America

## Abstract

Few microbial functions have been compared to a comprehensive survey of the human fecal microbiome. We evaluated determinants of fecal microbial β-glucuronidase and β-glucosidase activities, focusing especially on associations with microbial alpha and beta diversity and taxonomy. We enrolled 51 healthy volunteers (26 female, mean age 39) who provided questionnaire data and multiple aliquots of a stool, from which proteins were extracted to quantify β-glucuronidase and β-glucosidase activities, and DNA was extracted to amplify and pyrosequence 16S rRNA gene sequences to classify and quantify microbiome diversity and taxonomy. Fecal β-glucuronidase was elevated with weight loss of at least 5 lb. (*P* = 0.03), whereas β-glucosidase was marginally reduced in the four vegetarians (*P* = 0.06). Both enzymes were correlated directly with microbiome richness and alpha diversity measures, directly with the abundance of four Firmicutes *Clostridia* genera, and inversely with the abundance of two other genera (Firmicutes *Lactobacillales Streptococcus* and Bacteroidetes *Rikenellaceae Alistipes*) (all *P* = 0.05–0.0001). Beta diversity reflected the taxonomic associations. These observations suggest that these enzymatic functions are performed by particular taxa and that diversity indices may serve as surrogates of bacterial functions. Independent validation and deeper understanding of these associations are needed, particularly to characterize functions and pathways that may be amenable to manipulation.

## Introduction

The human intestinal microbial population is comprised of at least a trillion bacterial cells per gram of feces. [1] Most of these bacteria can be classified into 400–800 individual species, the majority of which are only known by sequence analysis. [1], [2] This gut microbial reactor is in close balance with the human host as it protects against pathogens, detoxifies potential poisons, produces energy and nutrients by digestion of food and de novo synthesis, conditions and maintains mucosal and systemic immunity, and facilitates homeostasis. [3], [4].

How the microbiota exerts these effects is starting to be understood using modern sequencing approaches, with which the genetic potential of a microbial community can be discerned. By using these culture-independent methods, researchers have started to obtain insight with respect to broad functional activities of the intestinal microbiota. [5]–[7] Yet, the interrelationships of genomic sequence analysis with specific bacterial functions and their role in host homeostasis is still poorly defined, in part because metagenomics only interrogates functional potential of a gene, not gene expression. Global assessment of microbial functions is not yet possible. However, advances can be made by studies of selected microbial enzymatic activities that are highly relevant to survival of the organism, as well as to disease in the human host.

Recent analysis of the gut microbiota using a deep sequencing approach has identified β-glucuronidase as a conserved function among bacteria colonizing the human gastrointestinal tract. [7] β-glucuronidases are believed to be an important and critical component in the cleavage of xenobiotic compounds and conjugated hormones that, once free, can re-enter the human body via enterohepatic circulation. The clinical relevance is that sustained systemic hormone levels via β-glucuronidase activity could contribute to hormone-sensitive pathologies such as the increased risk of breast cancer in postmenopausal women who have high endogenous estrogen levels. [8] On the other hand, recent studies suggest a hormone-related protection of colorectal cancer via enzymatic activation of phytoestrogens and other compounds found in fish oil, cruciferous vegetables, and estrogens. [9] β-glucosidases on the other hand, seem to have a more general role in the bioavailability of plant polyphenols and the extraction of energy from insoluble fibers and other indigestible carbohydrates. [10] Characterization of β-glucuronidases and β-glucosidases have shown that these important functions are selectively expressed by members of the gut microbiota and that diet composition partially explains the induction of genes expressing these enzymes. [11], [12] This suggests that the gut microbiota is a robust system that interconnects and is controlled by multiple factors and at multiple levels. A better understanding of the role of these enzymes in health and disease and their relationship with microbial diversity is needed.

In this study of healthy volunteers, we correlate functional activities of β-glucuronidase and β-glucosidase of the human gut microbiota with measures of microbial diversity and taxonomy. The goal is to identify microbial parameters that can provide insight on human-microbe homeostasis and that might serve as surrogates of bacterial functions.

## Results

In specimens provided by 51 volunteers (26 female, mean age 39, range 17–65 years), fecal β-glucuronidase and β-glucosidase activity levels were significantly correlated (R = 0.60, *P* = 0.0001). The enzyme activity levels had few significant associations with corresponding demographic or questionnaire parameters (Table 1). β-glucuronidase was 2.3-fold (e^0.85^) higher among participants who reported having lost at least 5 lb body weight in the previous year (*P* = 0.03). Weight loss was not related to β-glucosidase level, and neither enzyme was related to body mass index (BMI) overall. The four vegetarians had 0.57-fold (e^−0.56^) lower β-glucosidase levels (*P* = 0.06). Although antibiotics and other prescription medications used within the previous six months were strongly associated with fecal microbiome alpha diversity, [13] they were not associated with enzyme activities (Table 1).

**Table 1:** Associations of β-glucuronidase and β-glucosidase mean activity levels with demographic and questionnaire data of 51 study volunteers.

	β-glucuronidase^a^			β-glucosidase^a^	
*Variable (units)*	Beta	*P*-value		Beta	*P*-value
Gender (male = 0, female = 1)	0.32	0.22		0.20	0.22
Age (per year)	0.002	0.89		−0.01	0.09
Body mass index [BMI (per Kg/M^2^)]^b^	−0.03	0.49		−0.04	0.10
BMI category (<25 = 0, ≥25 = 1)	−0.05	0.81		−0.19	0.24
Weight gain (no change = 0, ≥5 lb = 1)	−0.12	0.73		−0.13	0.56
Weight loss (no change = 0, ≥5l b = 1)	0.85	0.03		0.28	0.26
Antibiotic use (<6 months = 1, else = 0)	−0.09	0.77		−0.19	0.30
Other prescription (<6 months = 1, else = 0)	0.04	0.89		−0.03	0.86
Food allergy (no = 0, yes = 1)	0.43	0.32		0.09	0.73
Vegetarian (no = 0, yes = 1)	−0.05	0.92		−0.56	0.06
Probiotic supplement use (n = 0, yes = 1)	−0.31	0.25		−0.26	0.13
Ever smoker (no = 0, yes = 1)	0.60	0.09		−0.04	0.84

a Beta values estimate the increase in log_e_ of enzymatic activity (IU/100 mg fecal protein) per unit increase in the independent variable.

b BMI models were adjusted for gender and age.

In contrast to the questionnaire data, both enzymes were directly and significantly correlated with four different measures of microbial richness and alpha diversity, indicating higher enzyme activity with higher microbial diversity (Table 2). Although all were statistically significant, the enzyme activities were less strongly associated with the alpha diversity measures that standardize for multiple copies of the same OTU (i.e., Chao1 and Shannon) and more strongly with counts of observed species (*P* = 0.01 for β-glucuronidase, *P* = 0.007 for β-glucosidase) and with phylogenetic distance (PD) whole tree (*P* = 0.002 for β-glucuronidase, *P* = 0.001 for β-glucosidase).

**Table 2:** Associations of alpha diversity measures and of major bacterial taxa and selected genera with levels of microbial enzymatic activity.

	β-glucuronidase^a^		β-glucosidase^a^	
	Beta^b^	*P*-value	Beta^b^	*P*-value
***Alpha diversity measures***				
Chao1	0.008	0.04	0.005	0.04
Shannon	0.661	0.02	0.414	0.02
Observed species	0.014	0.01	0.009	0.007
Phylogenetic distance (PD) whole tree	0.249	0.002	0.155	0.001
***Beta diversity measures*** c				
Principal component 1	−2.54	0.029	−1.75	0.012
Principal component 2	−3.53	0.003	−2.5	0.0005
***Major phyla, selected genera (mean relative abundance)***				
Firmicutes (80.3%)	0.002	0.932	−0.003	0.91
*Firmicutes Lactobacillales*				
* Streptococcaceae Streptococcus* (0.5%)	−0.2	0.04	−0.41	0.008
*Firmicutes Clostridia*				
non-*Clostridiales* (0.2%)	0.32	0.0001^d^	0.42	0.005
*Firmicutes Clostridia Clostridiales*				
* Lachnospiraceae Roseburia* (4.0%)	−0.26	0.02	−0.58	0.0001^d^
* Peptostreptococcaceae* genus NA (0.2%)	0.1	0.32	0.35	0.03
* Ruminococcaceae Oscillibacter* (1.2%)	0.24	0.01	0.19	0.23
* Ruminococcaceae Subdoligranulum* (2.8%)	0.28	0.002	0.53	0.0001
* Ruminococcaceae* genus NA (4.4%)	0.15	0.04	0.23	0.05
Bacteroidetes (16.9%)	−0.005	0.77	−0.0009	0.98
*Bacteroidetes Bacteroidales*				
* Rikenellaceae Alistipes* (0.6%)	0.3	0.003	0.56	0.0001^d^
Actinobacteria (1.3%)	0.002	0.27	0.002	0.55
Proteobacteria (0.5%)	−0.0002	0.78	−0.0002	0.84
Fusobacteria (0.2%)	−0.02	0.73	−0.06	0.49
Unclassified bacteria (0.8%)	0.09	0.32	0.28	0.055

a Log values of enzymatic activity IU/100 mg fecal protein. the two significantly associated principal components, per unit increase in alpha diversity, per tertile for Firmicutes and Bacteroidetes phyla, and per level (none, low, high) for the minor phyla, genera and unclassified bacteria.

b Beta values estimate the increase (or decrease for negative values) in loge of enzymatic activity for the two significantly associated principal components, per unit increase in alpha diversity, per tertile for Firmicutes and Bacteroidetes phyla, and per level (none, low, high) for the minor phyla, genera and unclassified bacteria.

c Principal component analysis based on unweighted Unifrac, adjusted for sex and age.

d Statistically significant at P<0.05 with Bonferroni correction for 61 taxa with mean relative abundance ≥0.1% that were evaluated.

Principal component (PC) analysis on the sample similarity matrix was used to derive 10 PC vectors as measurements of beta diversity. These PCs explained very little of the variance. Nonetheless, the first PC (7% of variance) and especially the second PC (6% of variance), but none of the other PCs, were significantly associated with reduced levels of both enzymes (Table 2). The Shannon index was significantly reduced with the second PC (R = −0.71) but not with the first PC (R = −0.08); correlations with the other measures of alpha diversity were very similar (Results not presented).

Turning to taxonomy, we examined 61 taxa with a mean relative abundance ≥0.1%, including bacteria in 5 defined phyla, 55 genera, and an unclassified group. The activities of one or both enzymes were associated with the relative abundance of eight genera, but they were not associated with the two major or three minor phyla (Table 2). Inverse associations, especially for β-glucosidase, were found with the abundance of two Firmicutes, *Streptococcus* and *Roseburia*. Activities of both enzymes were directly associated with the abundance of *Alistipes* in the phylum Bacteroidetes and also with the abundance of five *Clostridia* genera, especially *Ruminococcaceae, Subdoligranulum* and non-*Clostridiales* (Table 2). The taxa associated with high enzyme activities appeared to be under-represented in the second PC (R = −0.45 to −0.73).

## Discussion

We examined how the activities of two fecal microbial enzymes related to demographics of study participants, simple dietary factors, antibiotics and other medications, and especially to diversity and taxonomy of the distal human gut microbiome. Our major finding was that the activities of both enzymes were directly correlated with increased alpha diversity and especially with increased richness of the fecal microbiome. This complements our finding that the enzyme activities were associated with the abundance of a few relatively rare bacterial taxa, but not with the abundance of the major bacterial phyla. Likewise, in our beta diversity analysis, enzyme activities were significantly reduced in two distinct microbial communities, one of which (PC2) was sparsely populated with the high-enzyme activity taxa. Not withstanding evidence of *in vitro* β-glucosidase activity of several cultured strains of Firmicutes and Bifidobacteria in the phylum Actinobacteria, [11] our findings suggest that β-glucuronidase and β-glucosidase enzymatic functions in our participants were mostly performed by particular genera rather than by the microbial community at large.

Activities of both enzymes were directly associated with higher abundance of five *Clostridia* genera in the phylum Firmicutes, especially with *Ruminococcaceae, Subdoligranulum* and non-*Clostridiales*, largely complementing and corroborating *in vitro* results, such as the previously noted high prevalence of the classical *gus* gene and β-glucuronidase activity among *Ruminococcaceae* isolates. [11], [12], [14], [15] Comparison of our results to the *in vitro* studies is difficult, but some differences should be noted. [7], [11] We found that activity levels of both enzymes had significant inverse associations with the relative abundance of *Roseburia*, whereas Dabek and colleagues noted that all eight *Roseburia* strains examined produced β-glucosidase and six of these also produced β-glucuronidase. [11] Moreover, Gloux et al found one *Roseburia* strain that harbored a newly discovered class of genes with β-glucuronidase (BG) activity. [7] Perhaps of interest, this class of BG genes also was found in strains of *Ruminococcaceae Subdoligranulum*, [7] a genus for which relative abundance was directly correlated with the activity levels of both enzymes in our study. Enzyme activities in our study also were directly associated with the abundance of *Alistipes* in the phylum Bacteroidetes, which did not harbor the newly discovered BG genes, but which also has not been evaluated for *in vitro* production of β-glucuronidase or β-glucosidase. [11], [12] GenBank notes that the complete genome of *Alistipes shahii* WAL 8301 (Accession number FP929032), a gut isolate, contains seven copies of a BG gene. Further annotation, functional and metatranscriptomic approaches are clearly needed to discern whether the enzymatic levels detected *in vivo* correspond to a few highly active and readily inducible genes, or to more modest expression by multiple glycosidase genes acting in concert.

β-glucuronidase and other glycosidases are known to be expressed in a variety of microorganisms. In cultured specimens, Mroczynska and Libudzisz found that β-glucuronidase and β-glucosidase were produced at higher levels by *Lactobacillus* than by *Enterococcus* in fecal isolates that were obtained from people ages 1 to 80. [16] In environmental water specimens, β-glucuronidase was produced by several unidentified isolated gram-negative bacteria, by unidentified *Bacillus* species in the phylum Firmicutes, and by *Escherichia coli* (*E. coli*). [17] Several other Proteobacteria *Enterobacteriaceae* species, in addition to *E. coli*, can produce β-glucuronidase. [18] In our study, enzyme activities were not associated with *E. coli*, but this organism had a mean relative abundance of only 0.001%. At the phylum level, neither Proteobacteria nor Firmicutes correlated with the activities of β-glucuronidase or β-glucosidase.

Only two associations with our demographic and questionnaire data were noteworthy, perhaps suggesting that all human gut microbiota require these enzymatic functions. Our four vegetarians had lower β-glucosidase levels, resembling the observation of Ling and Hänninen, who reported that activities of both β-glucosidase and β-glucuronidase decreased within one week of changing from an omnivorous to an uncooked vegan diet. [19] Conversely, we noted significantly higher β-glucuronidase activity in participants who had lost weight. This observation is in agreement with a recent study reporting increase in β-glucuronidase activity in obese volunteers following a weight-loss diet. [12] Koning and colleagues saw an immediate decline in fecal β-glucuronidase activity during one week on amoxicillin, but this activity normalized within two months. [20] Thus, our null associations with antibiotic use within the previous six months are not surprising.

The primary limitations of our study are its small size, cross-sectional design, and lack of expression data or *in vitro* systems to explore the regulation and determinants of microbial enzymatic and other functions. It also was limited by the possibility that relatively rare taxa were misclassified with the Ribosomal Database Project due to sequencing errors, which precluded taxonomic comparisons below the genus level. Nonetheless, given the paucity of understanding, our study makes a useful contribution by demonstrating that β-glucuronidase and β-glucosidase activities are directly correlated with microbiome diversity and with particular genera. These findings suggest that these important functions are primarily accomplished by specialist organisms rather than by the community as a whole. If replicated and characterized in greater depth, perhaps the number of observed species could serve as a biomarker for these enzymatic functions, and ultimately particular strains or pathways might be exploited to improve health and reduce the risk of disease.

## Methods

### Participants and Ethics

We studied 51 employees of the Division of Cancer Epidemiology and Genetics, National Cancer Institute (NCI) who were recruited following approval by the NCI Special Studies Institutional Review Board and with signed informed consent. There were no exclusion criteria. In addition to collected specimens, a brief questionnaire was administered to all participants to gather general information on demographics, health, medication use and disease conditions that may affect the gut microbiota.

### Laboratory Methods

Specimen collection and assay methods are reported elsewhere. [13], [21] Briefly, from four fecal aliquots self-collected in RNAlater using two devices [Polymedco OC-auto collection device and Sarstedt fecal collection tube (Nümbrecht, Germany)], DNA was extracted and amplified by PCR targeting the V1–V2 region of the 16S rRNA gene. Pyrosequencing of the barcoded amplicons yielded 1,270,073 raw reads that were edited, de-noised, and assigned at 97% or greater similarity to a mean of 431 operational taxonomic units (OTUs) per participant. [13] OTUs from the four aliquots were averaged for the current analysis.

To assess an important function of the microbiota, deconjugation of glycosylated metabolites, β-glucuronidase and β-glucosidase activities were determined in triplicate by colorimetric enzyme kinetic assays in four replicates from the same stool that were independently collected by each participant in phosphate buffered saline. [21] These methods yielded highly reproducible estimates of alpha diversity, beta diversity, classification into the most abundant bacterial phyla, and functional activities of the two microbial enzymes. [13], [21] Because reproducibility of β-glucosidase activity with the Polymedco device was significantly lower than with the Sarstedt device, [21] the current analysis was restricted to the activities with the two Sarstedt devices, each run in triplicate, which were then averaged.

### Statistical Analyses

Alpha diversity measurements of the OTUs were generated with the QIIME pipeline (http://qiime.sourceforge.net). The sample similarity matrix was produced using unweighted UniFrac analysis in the QIIME pipeline. We then performed principal component (PC) analysis on the sample similarity matrix and derived 10 PC vectors as measurements of beta diversity. The sequences were assigned to taxa with the Ribosomal Database Project (RDP) naïve Bayesian classifier [22] and the Visualization and Analysis of Microbial Population Structures (VAMPS, Marine Biology Laboratories, Woods Hole, MA) pipeline.

Pearson’s correlation was used to assess the relationship between the activities of the two enzymes. The relationship between the two enzymatic activity levels and parameters of alpha diversity, beta diversity, and the questionnaire data [age, sex, body mass index (BMI), weight loss or gain, certain dietary characteristics, and use of antibiotics or other prescription medications] was estimated with Pearson’s correlation. Linear regression was used to test the association of enzymatic activity levels (average across triplicates with the paired Sarstedt devices, natural log-transformed) with multiple measures of alpha diversity and with relative abundance of 61 taxa, including phyla and selected genera [restricted to relative abundance ≥0.1% and ordered as zero, low (below median detected) and high (above median)]. Significance was based on trend tests or two-sided categorical tests with α = 0.05. Taxonomic associations with either enzyme that had nominal *P*≤0.05 are presented. Bonferroni was used to correct for 61 multiple comparisons.
